# On the origin of internal rotation in ammonia borane

**DOI:** 10.1007/s00894-014-2272-y

**Published:** 2014-05-27

**Authors:** Monika Parafiniuk, Mariusz P. Mitoraj

**Affiliations:** Department of Theoretical Chemistry, Faculty of Chemistry, Jagiellonian University, R.Ingardena 3, 30-060 Krakow, Poland

**Keywords:** Ammonia borane, Steric repulsion, Hyperconjugation

## Abstract

The internal rotation in ammonia borane (AB) was studied on the basis of natural orbitals for chemical valence (NOCV) and eigenvectors for Pauli repulsion (NOPR). We found that the total hyperconjugation stabilization (ca. 5 kcal mol^−1^), based on the charge transfer from the occupied σ (B–H) orbitals into the empty σ*(N–H), slightly favors the staggered conformation over the eclipsed one; however, the barrier to internal rotation in ammonia borane can be understood predominantly in a ‘classical’ way, as originating from the steric (Pauli) repulsion contributions (of the kinetic origin) that act solely between N–H and B–H bonds. Repulsion between the lone pair of ammonia and the adjacent B–H bonds was found to be dominant in absolute terms; however, it does not determine the rotational barrier. Similar conclusions on the role of CH↔HC repulsion appeared to be valid for isoelectronic ethane.

**Electronic supplementary material:**

The online version of this article (doi:10.1007/s00894-014-2272-y) contains supplementary material, which is available to authorized users.

**Figure:**
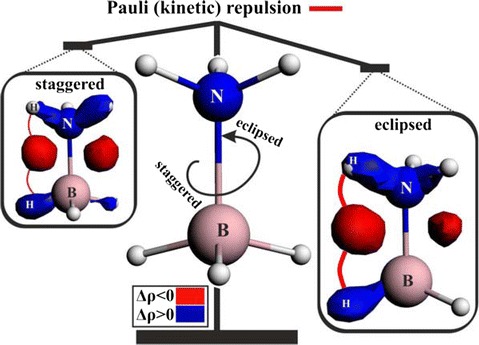
Pauli (kinetic) repulsion acting between the N-H and B-H bonds of ammonia borane

## Introduction

One of the most important goals of theoretical chemistry is to understand the origin of conformational changes in molecules [1, 2]. In order to achieve this goal many methods can be applied to the description of electronic structures: molecular orbitals (MOs) [3, 4], localized molecular orbitals (LMOs) [5–7], bond orders [8–17], atoms in molecules (AIM) [18], Fermi hole [19], kinetic energy and information theory based quantities [20–22], and various charge and energy decomposition schemes [23–28]. A useful and elegant approach suitable for description of energy profiles of chemical reactions was proposed by Torro-Labbe and coworkers [29] based on the reaction force concept. Bickelhaupt and Zeist [30] proposed the “activation strain model”, which also appears to be very useful in the analysis of chemical reactions.

Rotation around a single carbon–carbon bond is one of the most important conformational transitions in organic chemistry [31, 32]. A typical example is ethane, which exhibits staggered and eclipsed conformations; the former minimum energy structure is more stable than the transition state eclipsed structure by ∼3.0 kcal mol^−1^ [33]. The classical and intuitive explanation of the barrier suggested in organic textbooks is based on the steric repulsion between C–H bonds, which is dominant in the eclipsed structure [34]. An alternative explanation is based on hyperconjugation stabilization, which is stronger in the staggered conformation [35–38]. However, as pointed out by Mulliken [35], hyperconjugation effect should have only a minor influence on the barrier. Bader et al. [39] stated that the barrier to rotation in ethane can be related to the polarization of charge density along the carbon–carbon bond. Goodman and coworkers [40] have shown, based on the natural bond orbitals (NBO) method, that ethane’s staggered conformation is the result of hyperconjugation. This point of view was also presented by Weinhold [41]. Goodman’s results based on the NBO method have been challenged by the work of Bickelhaupt and Baerends [42] based on the model of a chemical bond originating from fragmented molecular orbitals; according to these results the internal rotational barrier in ethane is due to Pauli repulsion acting between the CH bonds of opposite CH_3_ units. Subsequent calculations by Mo and coworkers [44, 45] and then by Pendas et al. [43], confirmed the classical, steric-based interpretation of the barrier; in addition, the former authors have shown that hyperconjugation stabilizes the staggered conformer only by about 4 kJ mol^−1^ relative to the eclipsed form [45]. A very elegant recent paper by Mo and Gao [46] provided a compact overview of the most important studies on this subject; the main conclusion is that the internal rotational barrier in ethane is due predominantly to steric effects acting between C–H bonds, with minor participation stemming from hyperconjugation.

We have recently developed the ETS-NOCV scheme [47–50] by combining the extended transition state (ETS) [25, 26] energy decomposition approach with the natural orbitals for chemical valence (NOCV) method [51–57]. ETS-NOCV has proved suitable for qualitative and quantitative description of the crucial components (σ, π, δ, etc.) that constitute various types of chemical bonds [58–61]. In addition, the energy profiles of some chemical reactions can be also characterized [62, 63]. More importantly for this study, it was shown that NOCV representation allows for qualitative and quantitative description of hyperconjugation effects [47, 48]. Furthermore, the ETS energy decomposition scheme provides quantitative information on the Pauli repulsion effects [2, 25, 26].

Therefore, the main goal of this article was to apply for the first time the ETS-NOCV charge and energy decomposition scheme to analysis of the internal rotation in ammonia borane. Hyperconjugation and steric factors will be discussed in a detailed way. It should be noted that ammonia borane is considered nowadays as one of the most promising hydrogen storage materials. In addition, it was already proven that ammonia borane exhibits dissimilar features as compared to isoelectronic ethane [64]. Furthermore, the present study sheds additional qualitative and quantitative light on the steric repulsion in ammonia borane by decomposition of total Pauli repulsion into specific contributions stemming from different symmetry (σ and π). In order to achieve this goal, we defined for the first time the eigenvectors for Pauli repulsion; in this representation, one can thus discuss the Pauli repulsion components originating from different symmetries. For comparison, similar analyses will be performed for ethane.

### Computational details

All DFT calculations presented here were based on the Amsterdam Density Functional (ADF 2009.01) program [2, 65–68] in which the ETS-NOCV scheme was implemented [47–63]. The Becke-Perdew exchange-correlation functional [69, 70] was applied (BP86). A standard triple-zeta STO basis containing two sets of polarization functions (TZ2P) was adopted for all atoms. The contours of deformation densities were plotted based on ADF-GUI interface [71].

### Computational methods

Our analysis is based on the ETS-NOCV approach, which is a combination of the extended transition state (ETS) [25, 26] method with the natural orbitals for chemical valence (NOCV) scheme [51–57].

The basic concept of the ETS scheme involves partitioning of the total bonding energy Δ*E*_total_ between interacting fragments into four components:

1 $$ \varDelta {E}_{\mathrm{total}}=\varDelta {E}_{\mathrm{dist}}+\varDelta {E}_{\mathrm{elstat}}+\varDelta {E}_{\mathrm{Pauli}}+\varDelta {E}_{\mathrm{orb}} $$

The first component, Δ*E*_dist_, referred to as the distortion term, represents the amount of energy required to promote the separated fragments from their equilibrium geometry to the structure they will take up in the combined molecule; it can also be seen as strain energy. The second term, Δ*E*_elstat_, corresponds to the classical electrostatic interaction between the promoted fragments as they are brought to their positions in the final complex. The third term, Δ*E*_Pauli_, accounts for the repulsive Pauli interaction between occupied orbitals on the two fragments in the combined molecule. It is calculated as the difference between the energies of orthogonalized and non-orthogonalized fragments [2, 25]. Finally, the last stabilizing term, Δ*E*_orb_, represents the interactions between the occupied molecular orbitals of one fragment with the unoccupied molecular orbitals of the other fragment as well as the mixing of occupied and virtual orbitals within the same fragment (inner-fragment polarization). This energy term, Δ*E*_orb_, may be linked to the electronic bonding effect coming from the formation of a chemical bond (Eq. 2).

The NOCV are eigenvectors that diagonalize deformation density matrix ∆P^orb^ = P_molecule_ − P0, where P0 corresponds to the sum of density matrices for orthogonalized fragments; it has been shown that the natural orbitals for chemical valence pairs (ψ_-k_,ψ_k_) decompose the deformation density Δ*ρ*_orb_ into NOCV-contributions, *Δρ*_orb_^*k*^:

1 $$ \varDelta {\rho}_{orb}(r)={\displaystyle \sum_{k=1}^{M/2}{v}_k\Big[-{\uppsi}_{-k}^2(r)+{\uppsi}_k^2}(r)\Big]={\displaystyle \sum_{k=1}^{M/2}\varDelta {\rho}_{orb}^k} $$

where *ν*_k_ and *M* are the NOCV eigenvalues and the number of basis functions, respectively. Visual inspection of deformation density plots (*Δρ*_orb_^k^) helps to attribute symmetry and the direction of the charge flow. In addition, information gained from the analysis of deformation density plots can be enriched by providing the energetic estimations, *ΔE*_orb_^k^, for each *Δρ*_orb_^k^ within ETS-NOCV scheme:

2 $$ \varDelta {E}_{orb}={\displaystyle \sum_k\varDelta {E}_{orb}^k=}{\displaystyle \sum_{k=1}^{M/2}{v}_k\Big[-{F}_{-k,-k}^{TS}}+{F}_{k,k}^{TS}\Big] $$

where *F*_i,i_^TS^ are diagonal Kohn-Sham matrix elements defined over NOCV with respect to the transition state density (at the midpoint between density of the molecule and the sum of fragment densities). The above components *ΔE*_orb_^k^ provide the energetic estimation of *Δρ*_orb_^k^ that may be related to the importance of a particular electron flow channel for the bonding between the considered molecular fragments.

In the present study, in analogy to NOCVs, we defined for the first time the natural orbitals (eigenvectors) for Pauli repulsion, ϕ_k_, that diagonalize the Pauli deformation density matrix, ∆P^Pauli^ = P0−P_isolated_, where P_isolated_ is the sum of density matrices for non-orthogonalized fragments, whereas P0 correspond to the sum of density matrices for orthogonalized fragments. Such eigenvectors decompose the total Pauli deformation density, ∆ρ^Pauli^=ρ0 (orthogonalized-fragments)−ρ (non-orthogonalized-fragments), into the NOCV-like contributions (*Δρ*_k_^Pauli^) (in analogy to Eq. 1):

3 $$ \varDelta {\rho}^{Pauli}(r)={\displaystyle \sum_{k=1}^{N/2}{v}_k^{Pauli}\Big[-{\phi}_{-k}^2(r)+{\phi}_k^2}(r)\Big]={\displaystyle \sum_{k=1}^{N/2}\varDelta {\rho}_k^{Pauli}(r)} $$

The total charge transferred in this channel can be considered as:

4 $$ \varDelta {q}_k^{Pauli}={\nu}_k^{Pauli} $$

The present study characterized not only the total values of Pauli repulsion (Δ*E*_Pauli_) in ammonia borane based on the original ETS scheme (Eq. 1) but, in addition, provided a more detailed picture by analyses of both the Pauli repulsion contributions *Δρ*_k_^Pauli^ (Eq. 3) and the corresponding quantitative charge estimations *Δq*_k_^Pauli^ (Eq. 4). This approach (Eqs. 3, 4) was implemented by one of us in the home version of ADF2009.01. At present, the energetic Pauli repulsion contributions (*ΔE*_Pauli_^k^) from *Δρ*_k_^Pauli^ (calculated in an analogous way to Eq. 2) are unavailable. Hence, we focused our attention on the quantitative measures of *Δρ*_k_^Pauli^ based on Eq. 4. Red areas of deformation density channels correspond to charge depletion, whereas blue indicates charge accumulation upon bond formation.

Due to the fact that the steric interaction, which is a non-observable quantity [72], is very often attributed in the literature to Pauli repulsion quantum effect [2, 43, 46], we use both terms interchangeably throughout the text. Finally, we should note that Pauli repulsion is one of the bonding components in various energy decomposition schemes; hence, we believe that a more detailed description of this term based on Eqs. 3, 4, could be of wide interest. It is very important to point out that the main source of the Pauli repulsion is related to an increase in the kinetic energy contribution; so we could also refer to the Pauli repulsion term as kinetic repulsion due to the Pauli exclusion principle [2]. Such a concept, which relates the steric repulsion to the ‘kinetic energy pressure’ has already been put forward by various authors [73, 74]. In addition, the Pauli repulsion contribution appears to qualitatively correlate very well with the experimental Taft’s steric parameters [75].

## Results and discussion

**Fig. 1:**
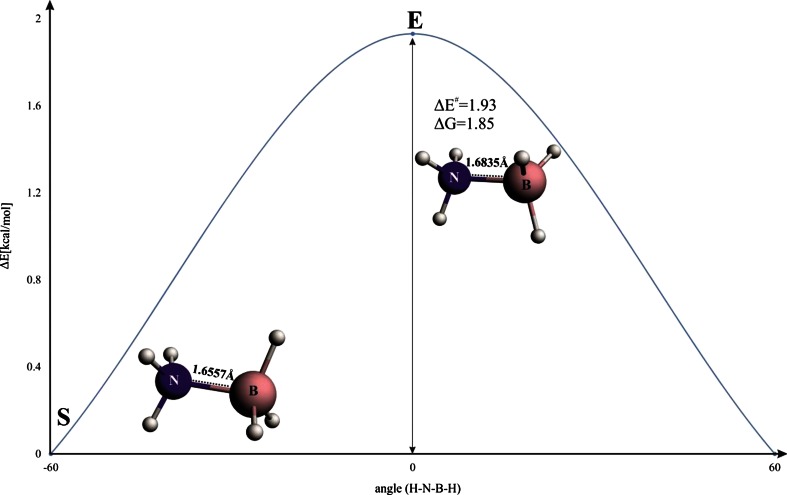
Energy profile for internal rotation in ammonia borane. B–N bond lengths (in Å) are indicated

**Fig. 2:**
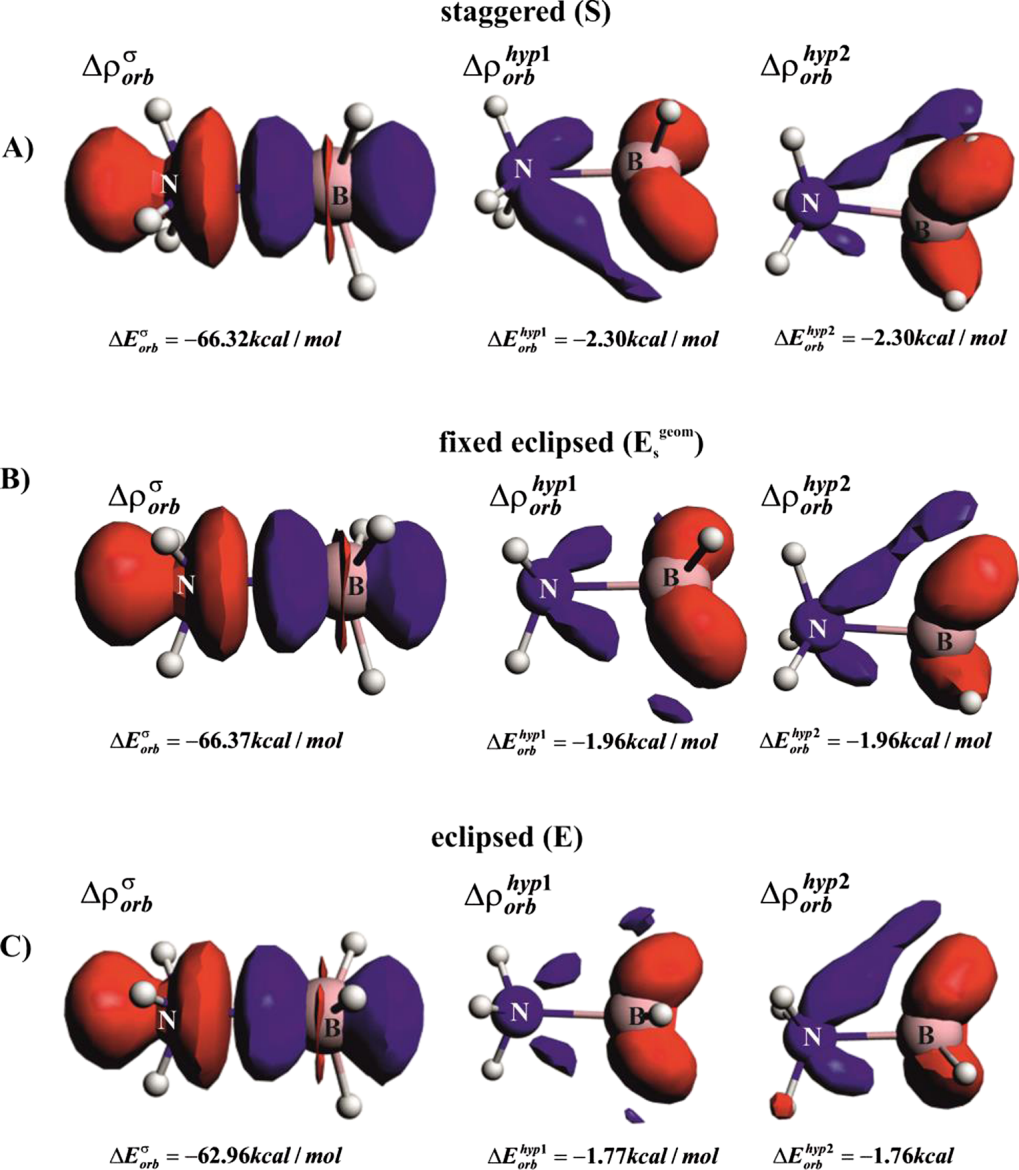
Dominant natural orbitals for chemical valence (NOCV)-based deformation density channels, *Δρ*_orb_^σ^, *Δρ*_orb_^hyp1^, *Δρ*_orb_^hyp1^, with the corresponding orbital interaction energies for the alternative isomers of ammonia borane. The contour value is |∆*ρ*| = 0.005 a.u. for *Δρ*_orb_^σ^, whereas for remaining hyperconjugation contributions 0.001 a.u. was applied. *Blue*/*red* contours correspond to accumulation/depletion of electron density. *S* Fully optimized staggered isomer, *E*_s_^geom^ eclipsed structure in the staggered geometry, *E* fully optimized eclipsed isomer

We will start with a brief description of the bonding situation in the most stable staggered conformation (**S**) of ammonia borane (Fig. 1). It can be seen from Table 1 that the bond dissociation energy (−Δ*E*_total_) amounts to 31.94 kcal mol^−1^ (BP86/TZ2P). This value fits well to the experimental enthalpy estimated by Haaland (31.1 ± 1 kcal mol^−1^ [76, 77]) as well as to other theoretical estimations [48, 78–81]. In line with previous studies [48, 78, 82–85], we found a slight dominance (by ∼0.7 kcal mol^−1^) of the electrostatic stabilization over the orbital interaction term (Table 1). Decomposition of the latter stabilizing term into NOCV-based deformation density channels leads to the conclusion that donation (*Δρ*_orb_^σ^) from the lone electron pair of ammonia to the lowest unoccupied orbital of BH_3_ is by far most dominant (*ΔE*_orb_^σ^ = − 66.32 kcal mol^−1^) as compared to the two hyperconjugation contributions, *Δρ*_orb_^hyp1^, *Δρ*_orb_^hyp2^; the corresponding orbital interaction stabilizations are *ΔE*_orb_^hyp1^ = *ΔE*_orb_^hyp2^ = − 2.30 kcal mol^−1^ (Fig. 2). The latter two degenerated contributions stem from charge transfer from the occupied σ (B–H) orbitals into the empty σ*(N–H) (Fig. 2). It is noteworthy that, in the isoelectronic ethane, the sum of stabilization arising from the two orthogonal hyperconjugation components was found to be significantly stronger (∼10 kcal mol^−1^) [46, 85].

**Table 1:** Extended transition state (ETS)^a,b^ energy decomposition results describing the H_3_N–BH_3_ bond in various isomers of ammonia borane. Charge estimates for Pauli repulsion contributions are indicated^c^

	*S*	*E*	*E*−*S*	*E*_s_^geom^
Δ*E*_total_	−31.94	−30.01	1.93	−29.87
Δ*E*_dist_	12.65	13.02	0.37	12.65
Δ*E*_elstat_	−77.32	−73.18	4.14	−77.8
Δ*E*_Pauli_	109.39	102.12	−7.27	111.5
Δ*E*_orb_	−76.66	−71.97	4.69	−76.22
*ΔQ*_global_^Pauli^(*Δq*_1_^Pauli^ + *Δq*_2_^Pauli^ + *Δq*_3_^Pauli^)	1.1833	1.1693	−0.014	1.2135
*Δq*_1_^Pauli^	0.7261	0.7049	−0.0212	0.7267
*Δq*_2_^Pauli^	0.2286	0.2325	0.0039	0.2434
*Δq*_3_^Pauli^	0.2286	0.2319	0.0033	0.2434

^a^∆*E*_total_ = ∆*E*_orb_ + ∆*E*_Pauli_ + ∆*E*_elstat_ + ∆*E*_dist_ [kcal mol^−1^]

^b^Labels assigned in Fig. 1; *E*_s_^geom^ corresponds to the eclipsed structure in the staggered geometry

^c^See Eqs. 3, 4 in Computational Methods and Fig. 5

**Fig. 3:**
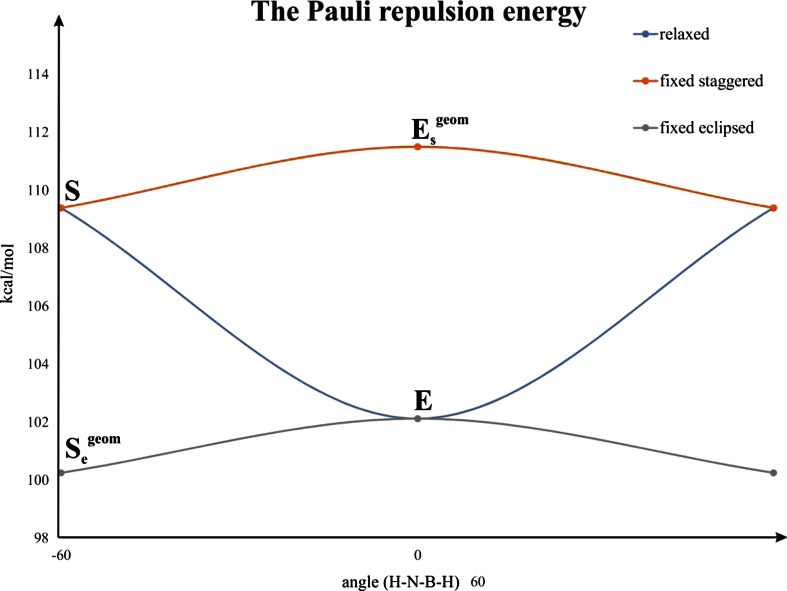
Pauli repulsion energies in the alternative isomers of ammonia borane. *Blue* Fully relaxed structures; *orange* staggered geometry; *gray* eclipsed geometry. *S* Fully optimized staggered isomer, *E*_s_^geom^ eclipsed structure in the staggered geometry, *S*_e_^geom^ staggered structure in the eclipsed geometry, *E* fully optimized eclipsed isomer

It is clear from Fig. 1 that rotation from the staggered to the eclipsed form leads to a change in energy, by 1.93 kcal mol^−1^. This barrier agrees quite well with the experimental value of 2.07 kcal mol^−1^ determined based on microwave spectra [86], and with other high level computations [87]. It is very important to point out that when going from the staggered (*S*) to the eclipsed isomer (*E*), one observes a notable stretch of the B–N bond, by ∼0.03 Å. Such elongation leads expectedly to a significant decrease in Pauli repulsion, by 7.27 kcal mol^−1^; at the same time the electrostatic (ΔE_elstat_) and orbital interaction (ΔE_orb_) contributions become less stabilizing, by 4.14 kcal mol^−1^ and 4.69 kcal mol^−1^, respectively (see Table 1 and the blue line in Fig. 3). From the examples of ethane [42] or biphenyl [88], it is known that this type of elongation when going from one isomer to the other is due to the steric (Pauli) repulsion. As indicated in a series of recent works [42–46, 88, 89], in order to estimate and characterize the forces leading to such elongation, one must first consider rigid rotation from the staggered to the eclipsed conformation; we have labeled such eclipsed conformation (in the staggered geometry) as *E*_s_^geom^. We can clearly see now from Table 1 and Fig. 3 (the orange curve), that an increase in the Pauli repulsion contribution, by 2.11 kcal mol^−1^, is noted when going from *S* to *E*_s_^geom^; it is important to note that the remaining bonding components are practically unchanged. A similar trend, i.e., the maximum Pauli repulsion in ammonia borane with the dihedral angle ∠(H–B–N–H) = 0.0, is noted when considering the rigid rotation from the geometry of the eclipsed structure to the staggered one (*S*_e_^geom^) (gray curve in Fig. 3). Thus, the Pauli (steric) repulsion contribution is responsible for stretching of the B–N bond and, accordingly, for the rotational barrier in ammonia borane; the analogous situation holds true for the ethane molecule, as demonstrated first by Bickelhaupt et al. [42] and then by others [43–46]. An increased kinetic repulsion (the main source of the Pauli term) in the *E*_s_^geom^ geometry is related through the virial theorem to the existence of repulsive forces acting predominantly on nitrogen and boron nuclei [18]. It must be added that hypercongutation stabilizations stemming from the charge transfer from the occupied σ (B–H) orbitals into the empty σ*(N–H) (*Δρ*_orb_^hyp1^, *Δρ*_orb_^hyp2^), favors the staggered conformation (Fig. 2), although the effect is minor (∼0.4 kcal mol^−1^) compared to changes in the remaining bonding contributions (Table 1). A quantitatively similar effect is observed for the change in the energy distortion contribution (Δ*E*_dist_) (Table 1).

**Fig. 4:**
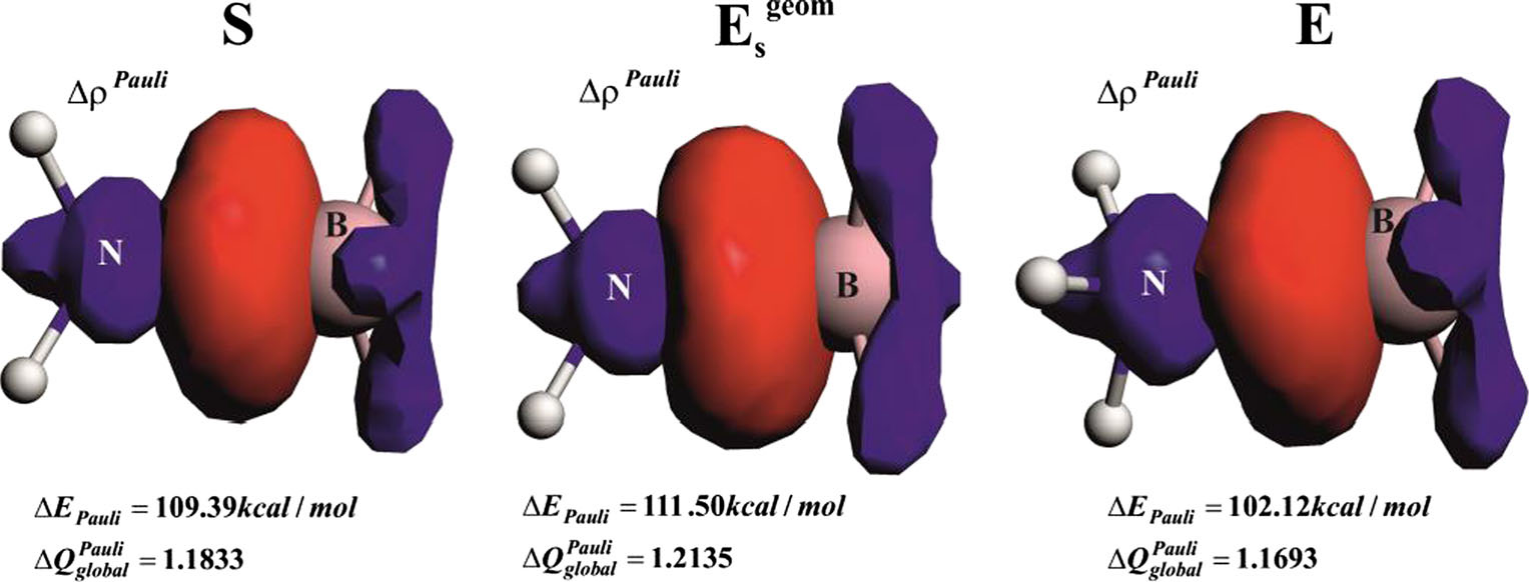
Contours of the total Pauli deformation density together with the corresponding energies. In addition, charge-based estimations are presented based on Eq. 4. The *blue*/*red* contours correspond to accumulation/depletion of electron density due to the Pauli exclusion principle

Let us now focus our attention on detailed changes in the Pauli repulsion contributions in the three ammonia borane isomers, *S*, *E*_s_^geom^ and *E*. Figure 4 presents the total Pauli deformation density contours (Δρ^Pauli^) together with the corresponding energy values (Δ*E*_Pauli_).

**Fig. 5:**
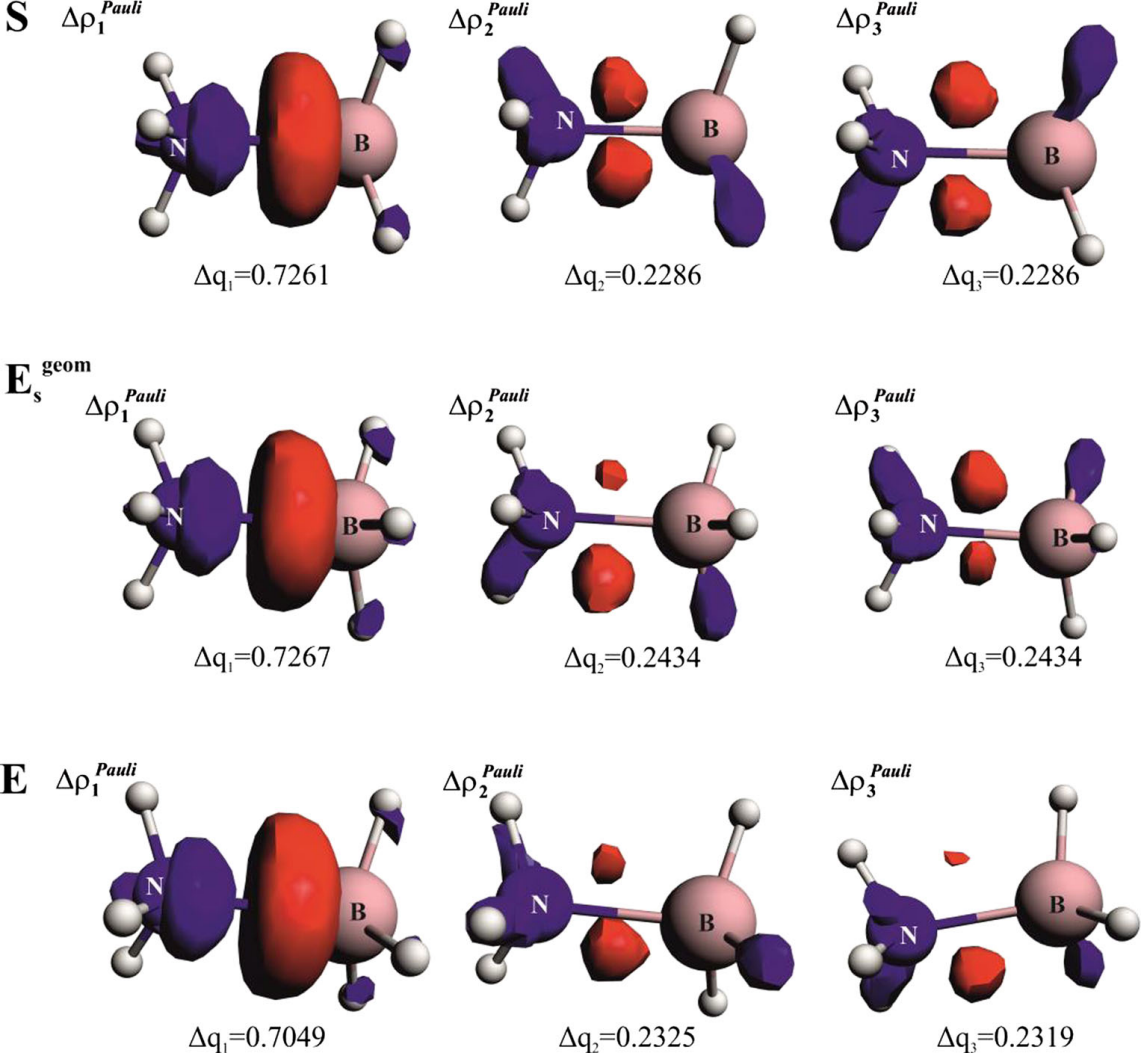
Dominant Pauli repulsion deformation density channels, *Δρ*_1_^Pauli^, *Δρ*_2_^Pauli^, *Δρ*_3_^Pauli^, together with the corresponding charge estimations, *Δq*_1_^Pauli^, *Δq*_2_^Pauli^, *Δq*_3_^Pauli^ in the selected ammonia borane conformations. The *blue*/*red* contours correspond to accumulation/depletion of electron density. *S* Fully optimized staggered isomer, *E*_s_^geom^ eclipsed structure in the staggered geometry, *E* fully optimized eclipsed isomer

As already stated, the *S*→ *E*_s_^geom^ transition leads to a jump in the Pauli term that it is then ‘relieved’ by elongation of the B–N bond in step *E*_s_^geom^→*E*. The important question that arises at this point is how the total Pauli repulsion is ‘distributed’ between NH_3_ and BH_3_ fragments. An analysis of function Δρ^Pauli^ leads to the observation that electrons are removed from the N–B binding region (in fact, it is a manifestation of the Pauli exclusion principle [2, 90]); although one can see that the red lobes extend also to the areas of NH↔HB interaction. However, such contours do not allow us to extract information on whether the total changes in Pauli repulsion are determined by the repulsive interaction between the lone electron pair of ammonia with the occupied σ (B–H) orbitals or directly by ‘classical’ N-H↔H-B repulsion [an interaction between the occupied σ (B–H) orbitals with the occupied σ (N–H)]. In order to obtain such separated information, we have decomposed total Pauli repulsion Δρ^Pauli^ into the contributions (*Δρ*_k_^Pauli^) according to Eq. 3. The three leading Pauli deformation density channels, *Δρ*_1_^Pauli^, *Δρ*_2_^Pauli^ , *Δρ*_3_^Pauli^, together with the corresponding quantitative charge estimations (Eq. 4) are presented in Fig. 5. It should be noted that the total charge, *ΔQ*_global_^Pauli^ = *Δq*_1_^Pauli^ + *Δq*_2_^Pauli^ + *Δq*_3_^Pauli^, that is removed from the H_3_N–BH_3_ binding region correlates well with the trend based on the Pauli repulsion energy (Table 1, Fig. 4). Qualitative inspection of the contours *Δρ*_i_^Pauli^ leads to the important observation that the first channel (*Δρ*_1_^Pauli^) corresponds solely to the interaction between the lone electron pair of ammonia with the B–H bonds, whereas the two latter orthogonal contributions (*Δρ*_2_^Pauli^, *Δρ*_3_^Pauli^) show NH↔HB repulsion (Fig. 5). More importantly, quantitative analysis of the charge depletion, based on the eigenvalues (Eq. 3), leads to the conclusion that, when going from *S*→ *E*_s_^geom^, the major changes (by 0.0148 a.u.) are within the second and third values of *Δq*_2_^Pauli^, *Δq*_3_^Pauli^. The repulsion *Δρ*_1_^Pauli^ characterized by *Δq*_1_^Pauli^ remains unchanged. Once going to the relaxed eclipsed structure, the Pauli contribution is further ‘relieved’ (*Δρ*_i_^Pauli^ values decrease in line with ΔE_Pauli_). These results show that an increase in the total repulsion in the eclipsed conformation compared to staggered (*S*→ *E*_s_^geom^) is determined solely by the NH↔HB repulsion (of the kinetic origin) due to the Pauli exclusion principle (an interaction between the electrons with the same spin as within the B–H and N–H bonds). The repulsive contribution from the interaction between the lone electron pair of ammonia with the electrons of B–H bonds (*Δρ*_1_^Pauli^) is dominating in absolute terms; however, it does not influence the barrier. These results confirm the ‘classical’ view that the internal rotational barrier in ammonia borane can be understood solely in terms of NH↔HB steric (Pauli) effects, with minor participation stemming from the hyperconjugation (Fig. 2) and geometry distortion term. It must be further noted that we performed a detailed study of the changes in *Δq*_i_^Pauli^ values (based on various sets of molecules) and have found that differences in the second decimal place are quantitatively meaningful.

Finally, we performed similar ETS-NOCV and Pauli repulsion analyses for ethane and found that CH↔HC Pauli (kinetic) repulsion channels are responsible for the rotation of methyl groups (see Supplementary electronic material); this is in line with the conclusions reached first by Bickelhaupt and subsequently by other researchers [42–46]. It is important at this point to cite another important work in the field explaining the origin of rotation in ethane. It is possible to decompose the barrier into changes in the kinetic (ΔT) and potential energy contributions: nuclei–-nuclei (ΔV_nn_), nuclei–-electrons (ΔV_ne_) and electrons-electrons (ΔV_ee_), as done by Bader and others for ethane [18]. Such an approach, while very physical and compelling, does not allow, for example, separate discussion of the role of the hyperconjugation effect, which is well rooted in chemistry. It was shown that rigid rotation *S*→ *E*_s_^geom^ leads to a rise in all of the destabilizing terms (ΔT ∼ +9 kcal mol^−1^, ΔV_nn_+ΔV_ee_ ∼ +16 kcal mol^−1^), whereas the electron-nuclei stabilization is ΔV_ne_ ∼ −22 kcal mol^−1^. (Fig. 6.11 in [18]). At this non-equilibrium geometry, the rise in kinetic energy is related, through the virial theorem, to the repulsion force acting on the nuclei. Accordingly, in the next step, *E*_s_^geom^→*E*, the CC bond elongates, which leads to weakening of the attraction (ΔV_ne_ ∼ +207 kcal mol^−1^) and decrease in the repulsion (ΔV_nn_+ΔV_ee_ ∼ –201 kcal mol^−1^; ΔT ∼ –3 kcal mol^−1^, the values are provided with respect to ethane in staggered geometry). One should also note that various authors have combined the above contributions in different ways [31]. Finally, Liu and Govind [91], defined in an elegant way at DFT level, the steric contribution (equal to the kinetic Weizsäcker term) from a difference between the total electronic energy and the sum of electrostatic (ΔV_ne_+ΔV_ee_+ΔV_nn_) and quantum energy terms ΔE_q_ (comprising the sum ΔE_xc_+ΔE_Pauli_); the change in the kinetic term due to the Pauli exclusion principle is incorporated in Δ*E*_Pauli_. It was shown that rigid rotation *S*→ *E*_s_^geom^ results in the appearance of destabilizing forces originating from the fermionic quantum contribution Δ*E*_q_; closer inspection of the author’s data shows that this change is due entirely to a rise in the kinetic energy term [91]. Finally, one should cite the separate work of Nagy [92], who discussed the Fisher information based on the kinetic term; the role of kinetic energy and the information origin of the chemical bonding have been studied by Nalewajski [22].

## Concluding remarks

The present work studied for the first time the internal rotation in ammonia borane based on our recently developed charge and energy decomposition scheme, ETS-NOCV, as well as the eigenvectors for Pauli repulsion. Detailed analyses of the electronic and the steric factors were performed in order to understand the origin of the barrier to rotation in ammonia borane.

We found that the barrier to rotation, staggered ↔ eclipsed, is only ∼2 kcal mol^−1^. It was demonstrated using the ETS-NOCV scheme that the hyperconjugation, originating from the charge transfer from the occupied σ (B–H) orbitals into the empty σ*(N–H), favors the staggered isomer, although, quantitatively it leads to only a slight stabilization (∼5 kcal mol^−1^). For ethane, this stabilization was more pronounced, ∼10 kcal mol^−1^. We have found, based on our newly proposed scheme, the natural orbitals for Pauli repulsion, that rigid rotation from the staggered to the eclipsed conformation causes predominantly the enhancement of steric (Pauli) repulsion acting solely between N–H and B–H bonds; this is subsequently ‘relieved’, leading to elongation of the B–N bond in the fully optimized eclipsed structure. Analogous trends were found for ethane (Table S1). Accordingly, the barrier to rotation in ammonia borane can be understood in a classical way; namely, as originating from the steric (Pauli) repulsion contributions that act solely between N–H and B–H bonds. Repulsion between the lone pair of ammonia and the B–H bonds is dominant in absolute terms; however, it does not influence the barrier.

## Electronic supplementary material

Below is the link to the electronic supplementary material.

ESM 1 (DOC 75 kb)
