# Supplementary material for: On the origin of internal rotation in ammonia borane
Source: J Mol Model. 2014 May 27;20(6):2272. doi: 10.1007/s00894-014-2272-y (PMC4072093; doi:10.1007/s00894-014-2272-y)
Supplement: Supplementary file 1 — (DOC 75 kb) [file 894_2014_2272_MOESM1_ESM.doc]

***Supporting Information***

*To be considered for publication in the Special Issue of Journal of Molecular Modeling from MIB’13*

**On the Origin of Internal Rotation in Ammonia Borane**

Monika Parafiniuk, Mariusz P. Mitoraj^*^

Department of Theoretical Chemistry. Faculty of Chemistry. Jagiellonian University. R.Ingardena 3. 30-060 Krakow. Poland.

**Table S1.** ETS-energy decomposition scheme describing the bond between methyl units in various isomers of ethane. In addition quantitative charge estimates of Pauli repulsion contributions are presented (Δ*q*_i_^Pauli^).

|  | **S** | **E** | **E_s_^geom^** |
| --- | --- | --- | --- |
| **ΔE_total_** | -93.69 | -91.08 | -91.03 |
| **ΔE_dist_** | 21.1 | 22.22 | 21.10 |
| **ΔE_elstat_** | -131.41 | -126.95 | -131.73 |
| **ΔE_Pauli_** | 200.79 | 192.92 | 203.36 |
| **ΔE_orb_** | -184.17 | -179.27 | -183.76 |
|  |  |  |  |
| ^1)^$\Delta q_{1}^{Pauli}$ | 0.4510 | 0.4427 | 0.4512 |
| ^2)^$\Delta q_{2}^{Pauli}$ | 0.1299 | 0.1333 | 0.1375 |
| ^2)^$\Delta q_{3}^{Pauli}$ | 0.1299 | 0.1332 | 0.1374 |

1. Pauli repulsion term describing interaction between the SOMO of methyl units with the occupied σ(C-H) orbitals.
2. Pauli repulsion contribution describing solely CH↔HC interactions.
